# NK Cells and γδ T Cells Mediate Resistance to Polyomavirus–Induced Tumors

**DOI:** 10.1371/journal.ppat.1000924

**Published:** 2010-05-27

**Authors:** Rabinarayan Mishra, Alex T. Chen, Raymond M. Welsh, Eva Szomolanyi-Tsuda

**Affiliations:** Department of Pathology, University of Massachusetts Medical School, Worcester, Massachusetts, United States of America; University of Michigan, United States of America

## Abstract

NK and γδ T cells can eliminate tumor cells in many experimental models, but their effect on the development of tumors caused by virus infections in vivo is not known. Polyomavirus (PyV) induces tumors in neonatally infected mice of susceptible strains and in adult mice with certain immune deficiencies, and CD8+ αβ T cells are regarded as the main effectors in anti-tumor immunity. Here we report that adult TCRβ knockout (KO) mice that lack αβ but have γδ T cells remain tumor-free after PyV infection, whereas TCRβ×δ KO mice that lack all T cells develop tumors. In addition, E26 mice, which lack NK and T cells, develop the tumors earlier than TCRβ×δ KO mice. These observations implicate γδ T and NK cells in the resistance to PyV-induced tumors. Cell lines established from PyV-induced tumors activate NK and γδ T cells both in culture and in vivo and express Rae-1, an NKG2D ligand. Moreover, these PyV tumor cells are killed by NK cells in vitro, and this cytotoxicity is prevented by treatment with NKG2D-blocking antibodies. Our findings demonstrate a protective role for NK and γδ T cells against naturally occurring virus-induced tumors and suggest the involvement of NKG2D-mediated mechanisms.

## Author Summary

Virus-induced tumors account for a large fraction of malignancies in both humans and mice. These tumors express viral antigens and have been thought to be controlled mostly by αβ TCR+ CD8 T lymphocytes that are specific for viral peptides. We found that mice lacking αβ T cells are protected from the formation of tumors induced by the small DNA virus polyoma (PyV) if they have γδ T and NK cells. Moreover, cell lines we established from the virus-induced tumors induced NK and γδ T cell activation, and expressed Rae-1, a cellular stress molecule which serves as ligand for NKG2D, an activating receptor on NK and γδ T cells. NK and γδ T cells seemed to mount antitumor but not antiviral responses, as their presence did not change the amount of persisting virus significantly. Our studies suggest that mice have a multipronged host defense against PyV-induced tumors that includes γδ T and NK cells in addition to αβ T cell responses. Merkel cell virus, a tumor causing polyomavirus in humans, is closely related to PyV with a similar biology, making it very important to understand mechanisms involved in host control of tumor development in the course of these life-long persistent infections.

## Introduction

Virus-induced tumors mostly develop in immune-compromised hosts, suggesting that the immune system provides protection against the induction and/or progression of these tumors. T cells expressing α and β TCR and recognizing viral peptide epitopes are thought to be important for this protection. However, other cell types of the immune system, including NK cells and γδ T cells, are also endowed with effector functions similar to those of αβ T cells, but their role in the control of virus-induced tumors is largely unexplored.

A growing body of experimental evidence suggests that tumor cells can be recognized and eliminated by NK cells and γδ T cells. In a variety of human cancers such as lung, colon and renal cell carcinomas NK cells and γδ T cells can be found among tumor infiltrating lymphocytes (TIL) [1], [2], [3], [4]. Moreover, NK cell infiltration of tumors was noted to be associated with improved prognosis in some human cancers [4], [5], [6]. Implanted syngeneic tumors, including those induced by tumor viruses, grow more aggressively in mice if no functional NK cells are present [7]. γδ T cells can also protect mice against transplanted hematopoietic tumors [8], and mice deficient in γδ T cells have an increased susceptibility to chemically induced cutaneous tumor formation [9]. Acute virus infections, as well as other NK cell activating agents, can augment the rejection of implanted tumor cells [7]. Nevertheless, evidence that NK and γδ T cells can control the formation and progression of naturally occurring virus-induced tumors is lacking.

Polyomavirus (PyV), a small DNA tumor virus that carries potent oncogenes, can transform a variety of cells in culture readily, but infection of adult immune competent mice (the natural host for PyV) does not lead to tumor formation. However, PyV infection causes a wide variety of tumors affecting multiple tissues and cell types when neonatal mice of some “susceptible” mouse strains are infected, and it also causes tumors in adult mice with certain immune-deficiencies [10], [11]. Neonatal mice of the tumor susceptible mouse strains rapidly gain resistance after birth, and become refractory to tumor induction by the virus within a few days. The importance of the immune system in tumor resistance is indicated by observations that mouse strains highly resistant to PyV-induced tumor formation could be rendered tumor susceptible with immune suppressive treatments such as neonatal thymectomy, irradiation, and administration of anti-lymphocyte serum [12], [13], [14].

A high level of virus replication and spread seems to be a prerequisite for PyV-induced tumor development. Therefore, antiviral immune responses which decrease the virus load and reduce the levels of virus persistence may also decrease the chances of tumor formation. This does not mean, however, that antiviral resistance is always coupled with resistance against tumors and vice versa. For example, antibody responses to PyV reduce the virus load, but they do not prevent tumor formation [15]. CD8 T cells specific for PyV viral peptides, on the other hand, reduce virus load and also have a role in preventing the formation of virus-induced tumors [16], [17], [18]. Endogenous super-antigens encoded by a mouse mammary tumor provirus Mtv-7 have been shown to increase susceptibility of neonatal mice to PyV-induced tumors by eliminating Vβ6+ T cells from their T cell repertoire; Vβ6T cells make up the majority of the CTLs reactive to the dominant middle T peptide epitope in H2k mice [19]. Of note is that in the rejection of PyV-induced tumor cells transferred into PyV-immune mice both CD4 and CD8 αβ T cells were shown to play a role [20]. Consistent with the role of αβ T cells mediating tumor resistance, athymic nude mice infected as adults developed tumors, as did mice lacking β2m, an essential component of MHC class I molecules [17]. However, it is possible that there is a redundancy in antitumor responses and that CD8 T cells specific for viral peptides do not act only by themselves against the PyV-transformed tumor cells. The contribution of various other cell types capable of cytotoxic activity, such as γδ T cells or NK cells to PyV tumor resistance in vivo has not been investigated so far.

Here we report that γδ T and NK cells can prevent tumor formation in PyV-infected αβ T cell-deficient mice. PyV-induced tumor cell lines activate γδ T cells and NK cells in vitro and in vivo. Moreover, these tumor cell lines express Rae-1 ligands recognized by NKG2D, an activating receptor expressed on all NK cells and some γδ T cells, they are efficiently killed by NK cells in vitro and this killing is NKG2D-mediated. These data taken together show a major role for γδ T cells and NK cells in tumor resistance in a naturally occurring virus-induced tumor model, implicate NKG2D-NKG2D ligand interactions in these antitumor responses, and suggest that αβ T cells, γδ T cells and NK cells may work together to prevent tumor formation during life-long virus persistence.

## Results

### PyV infection induces a high incidence of tumor development in TCRβ×δ KO and E26 (T and NK cell-deficient), but not in TCRβ KO mice

**Figure 1:**
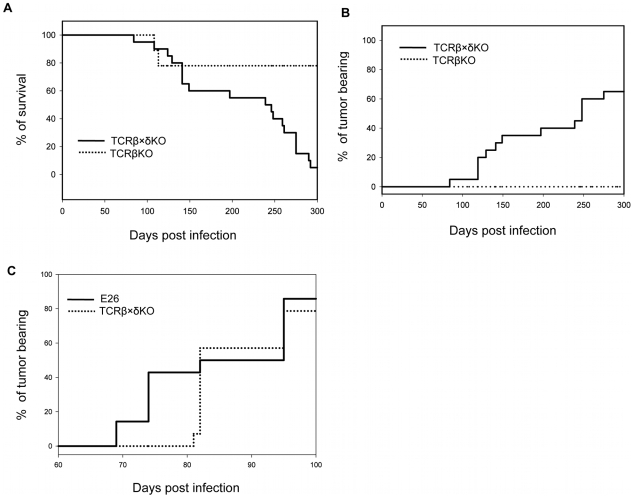
Survival and tumor incidence of PyV-infected TCRβ KO, TCRβ×δ KO and E26 mice. (A) Survival of TCRβ KO and TCRβ×δ KO mice after PyV (2×10^6^ p.f.u., i.n.) infection. (B) Tumor incidence in TCRβ KO and TCRβ×δ KO mice in the same experiment. N = 20 mice per group. (C) Tumor incidence in E26 mice that lack both NK and T cells and in TCRβ×δ KO mice after PyV (2×10^6^ p.f.u., i.p.) infection. N = 15 mice per group. One representative of at least two similar independent experiments is shown.

In order to understand which components of the immune system are required to control PyV -induced tumor formation in adult mice we tested the outcome of long-term PyV infection in TCRβ KO mice that lack αβ T cells, TCRβ×δ KO mice that lack all αβ and γδ T cells, and E26 mice that lack all T cells and NK cells [21]. Following intranasal infection with 2×10^6^ p.f.u. of PyV strain A2 (“high tumor” strain) ∼80% of TCRβ KO mice survived for ten months and all of them were free of tumors, whereas only 1 out of 20 (5%) TCRβ×δ KO mouse was alive at this time point and ∼60% of the TCRβ×δ KO mice had developed large tumors (Fig. 1A and B). The tumors were detected by visual observation and palpation, usually when their size exceeded ∼0.5 cm in diameter. The TCRβ×δ KO mice which died but are not counted as tumor bearing had hind leg paralysis, a condition seen in many previous studies [22], [23] and likely due to bone tumors of the spine or possibly other PyV-induced neurological conditions, or died of unknown causes. The tumors found by gross examination were almost all salivary gland tumors, often occurring bilaterally. The lack of a wide spectrum of tumors in these mice is similar to findings previously reported for B6/β2m KO mice infected as adults [22]. Similar results were obtained with mice infected intraperitoneally (i.p.). For example, in a more limited study, 0/8 TCRβ×δ KO mice, 4/5 TCRβ KO mice and 10/10 B6 mice were alive and tumor-free at 5 months post infection when PyV was given i.p. There was an earlier appearance of tumors in E26 mice than in TCRβ×δ KO mice (Fig. 1C), suggesting that NK cells may also contribute to tumor resistance and delay the onset of tumor formation.

### PyV load is not different in organs of TCRβ KO and TCRβ×δ KO mice

**Figure 2:**
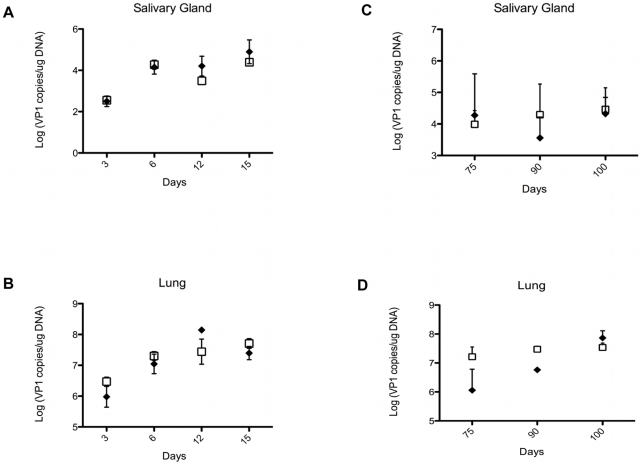
Viral load in organs of TCRβ KO and TCRβ×δ KO mice at different time points after in PyV infection. The viral load was determined by qPCR measuring PyV genome copies per µg of organ DNA in samples isolated from salivary glands (A, C) and lungs (B, D) at various time points during acute (A, B) and long-term persistent infection (C, D). N = 3 in (A) and (B), N = 5 in (C) and (D). Open squares: TCRβ×δ KO mice, closed diamonds: TCRβ KO mice, mean +/− s.d. One representative of three independent experiments is shown.

NK cells and γδ T cells may provide protection against PyV-induced tumors by directly eliminating the emerging tumor cells, or indirectly, by lowering the PyV titer in various organs and thereby decreasing the chances of cell transformation and tumor development. To test the effect of γδ T cells on viral load we compared the number of PyV genome copies in the salivary glands and lungs of TCRβ KO and TCRβ×δ KO mice at various time points after intranasal PyV infection, but before tumors were detectable in TCRβ×δ KO mice. The number of viral genome copies on days 3, 6, 12 and 15 (Fig. 2A and B; short-term infection) and on days 75, 90 and 100 (Fig. 2C and D; long-term infection), were not significantly different in the lungs or salivary glands between TCRβ KO and TCRβ×δ KO mice. This observation suggests that the γδ T cells provide protection against PyV-induced tumors by controlling tumor development, rather than by reducing viral load in the target organs prior to PyV-induced tumor formation. Previous studies in our lab showed that in vivo NK cell depletion did not increase PyV titers in the kidneys and spleens of SCID mice one week after PyV infection i.p., suggesting that NK cells did not have a direct role in the control of PyV levels in those organs at the acute phase of the infection [24].

### PyV-induced salivary gland tumor cell lines express the NKG2D ligand Rae-1

**Figure 3:**
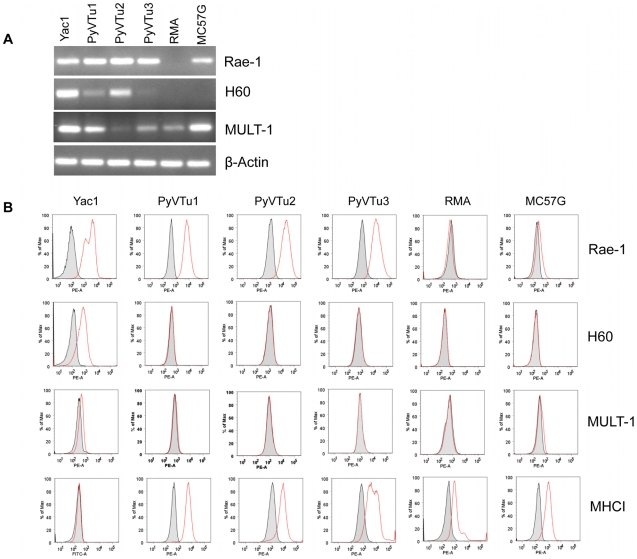
Expression of NKG2D ligands on cell lines established from PyV-induced salivary gland tumors of TCRβ×δ KO mice. (A) RT-PCR detection of Rae-1, H60, Mult1 and β-actin transcripts in three salivary gland tumor cells lines established from PyV-induced tumors that developed in TCRβ×δ KO mice (PyVTu1, PyVTu2 and PyVTu3), and in YAC-1, RMA and MC57G cell lines. The expression of message for β-actin in the same samples is shown at the bottom panel. (B) Expression of Rae-1, H60, Mult1 and MHC class I proteins on the surface of PyVTu1, PyVTu2 and PyVTu3 cell lines, and also on YAC-1, RMA and MC57G cells. Grey shaded area: staining with isotype controls, open red lines: Rae-1, H60, Mult1 or MHC I-specific antibody staining. The experiment was repeated two (for H60, MHC I) or more (for Rae-1) times with similar results.

NK cells and γδ T cells may recognize PyV-induced tumor cells by detecting tumor cell antigens on their surface via activating receptors, such as NKG2D. We established several cell lines from salivary gland tumors isolated from PyV-infected TCRβ×δ KO mice and tested them for the expression of the NKG2D ligands Rae-1, H60 and MULT1. These cell lines, PyVTu1, PyVTu2 and PyVTu3, were all positive for Rae-1, H60 and MULT1 transcripts by reverse transcriptase (RT) PCR (Fig. 3A). The Rae-1 specific primers could amplify cDNA reverse transcribed from mRNA of all Rae-1 family members, α, β, γ, δ and ε, and gave the same size PCR product. We also tested the expression of NKG2D ligand surface proteins on PyVTu cells by staining them with monoclonal antibodies against Rae-1, H60 and MULT1. All of the cell lines tested, PyVTu1, PyVTu2 and PyVTu3, had a high expression of Rae-1 but H60 or MULT1 surface protein expression was not detectable on these cell lines despite the abundant H60 and MULT1 transcripts (Fig. 3B). YAC-1 T cell lymphoma cells expressed Rae-1, H60 and MULT1 messages and surface proteins, whereas RMA T cell lymphoma cells did not express Rae-1, H60, only low levels of MULT1 mRNA, and none of these surface proteins, and served as negative controls. NK cells can also detect the expression of class I MHC molecules on their targets and preferentially kill cells displaying low levels of class I MHC molecules. However, we found that PyVTu cells express high levels of class I MHC molecules, unlike YAC-1 cells which have low levels of class I expression (Fig. 3B).

### PyVTu cells activate NK cells and γδ T cells in-vitro and in-vivo

**Figure 4:**
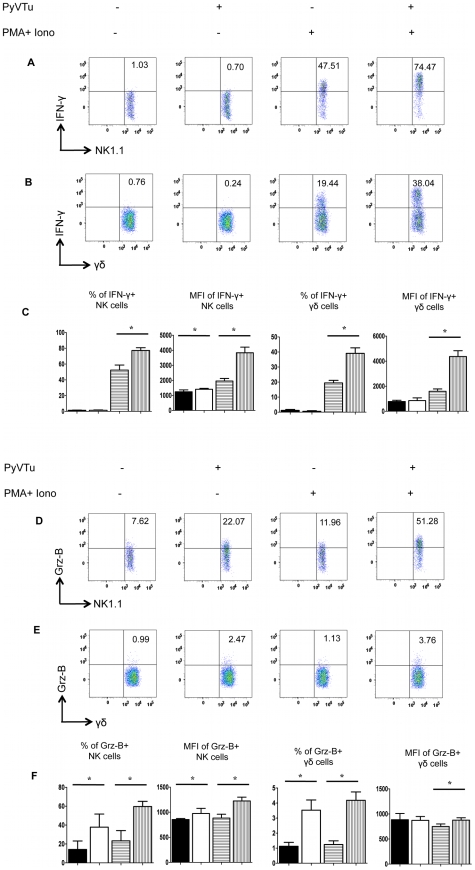
Activation of NK cells and γδ T cells by co-culture with PyVTu cells in vitro. (A) Flow cytometry of intracellular IFNγ staining of spleen cells cultured with or without PyVTu cells and/or PMA and ionomycin stimulation, gated on NK cells (NK1.1+CD3−) and (B) on γδ T cells (γδ TCR+CD3+). The numbers indicate the percentage of IFNγ+ NK or γδ T cells, respectively. (C) Percentages of IFNγ+ NK and γδ T cells and mean fluorescent intensity (MFI) of the IFNγ staining obtained with cultured spleen leukocytes from three individual mice are summarized, mean +/− s.d. values are shown. The filled bars indicate cultures without PyVTu cells and stimulation, the open bars with PyVTu cells, but without stimulation, the bars with horizontal stripes without PyVTu cells, but with stimulation and the bars with vertical stripes with PyVTu cells and PMA and ionomycin stimulation. The asterisks indicate statistically significant (P<0.05) differences determined by student's t test. (D) Intracellular granzyme-B (Grz-B) staining gated on NK cells and (E) γδ T cells in the same experiment. (F) Percentages of granzyme-B+ NK and γδ T cells and MFI of staining. Mean and s.d. of three cultures is shown, the bars are as described for (C). The asterisks indicate statistically significant (P<0.05) differences. The experiments shown were repeated at least twice with similar results.

To test the ability of PyVTu cells to activate NK cells and γδ T cells in vitro, spleen cells of uninfected TCRβ KO mice were incubated with PyVTu cells with or without PMA and ionomycin treatment, followed by intracellular cytokine staining for IFNγ and granzyme-B. PMA and ionomycin are commonly used for non-specific stimulation of cytokine production in various cell types and they were also reported to increase the IFNγ mRNA half-life in activated NK cells [25]. The PyVTu cells were incubated with spleen leukocytes at a ratio of 10∶1. Spleen cells from TCRβ KO mice were used in these experiments, because they contain a higher percentage of γδ T cells than do spleens of B6 mice. The addition of PyVTu cells and PMA and ionomycin treatment together resulted in a significant increase in the percentage of IFNγ producing NK cells and γδ T cells and also a major increase in the mean fluorescent intensity (MFI) of intracellular IFNγ staining in these cells compared to NK and γδ T cells only treated with PMA and ionomycin (Fig. 4A, B and C), suggesting that PyVTu cells played an important role in NK and γδ T cell activation. Incubation of NK cells and γδ T cells with PyVTu cells also led to increased granzyme-B production in these cell types compared to cultures without PyVTu cells, even in the absence additional PMA and ionomycin stimulation (Fig. 4D, E and F). The increase in granzyme-B production by γδ T cells following their co-culture with PyVTu cells was small, but reproducible, a consistent observation in multiple experiments.

**Figure 5:**
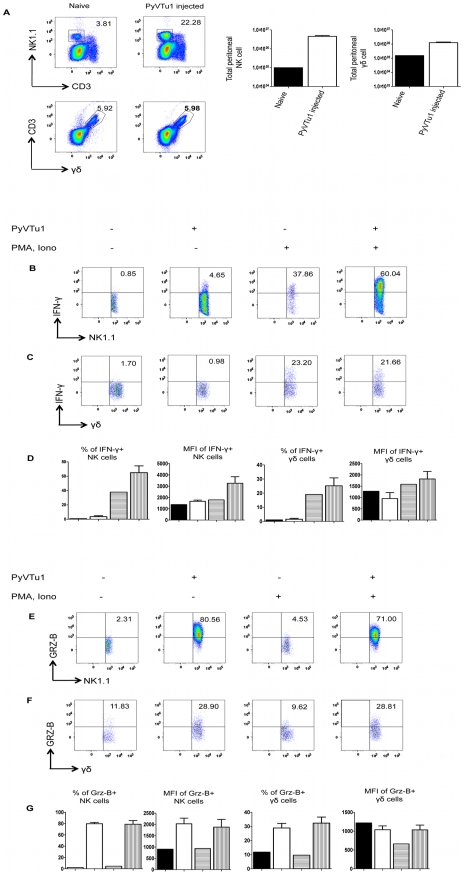
Activation of NK cells and γδ T cells in vivo by i.p. injection of PyVTu cells. (A) Percentages (left panel) and numbers (right panel) of peritoneal NK and γδ T cells in TCR β KO mice 3 days after i.p. injection of PyVTu1 (5×10^6^/mouse) cells. PECs of three naive mice that did not receive cells were pooled and analyzed in comparison, PECs from the PyVTu1 cell-injected mice were enumerated individually (n = 3), mean and +/− s.d. is shown. (B) Intracellular IFNγ staining of PEC gated on NK cells and (C) γδ T cells from untreated and PyVTu1 cell-injected TCRβ KO mice from the same experiment with or without PMA and ionomycin stimulation. The numbers indicate percentages of IFNγ + cells. (D) Percentages of IFNγ+ NK and γδ T cells and mean fluorescent intensity (MFI) of IFNγ staining. Filled bars represent pooled sample from 3 naïve mice without stimulation, open bars the mean + s.d. of samples from 3 PyVTu1-injected mice without stimulation, bars with horizontal stripes pooled samples from 3 naïve mice with stimulation and bars with vertical stripes the mean +s.d. of samples from 3 PyVTu1-injected mice with stimulation. (E) Intracellular Grz-B staining of NK and (F) γδ T cells in the same experiment. (G) Percentage of Grz-B+ NK and γδ T cells and mean fluorescent intensity (MFI) of staining. The bars are as described for (D). The experiment shown is one representative of at least 3 independent experiments.

We also tested whether PyVTu cells activate NK cells and γδ T cells in vivo. PyVTu cells were injected i.p. into TCRβ KO mice (5×10^6^ cells/ mouse), and 3 days later peritoneal exudate cells (PEC) were harvested and incubated for 4 hours with or without PMA and ionomycin stimulation, and tested for IFNγ and granzyme-B production by intracellular cytokine staining. There was an increase in the percentage of NK cells in the PEC population after injection of tumor cell lines. In one representative experiment 3 days after PyVTu cell injection there were 22.3% NK1.1+/ CD3− cells in the peritoneum, whereas control mice that did not receive PyVTu cells had approximately 3.8% NK cells (Fig. 5A). The total number of PEC recovered from mice three days after PyVTu cell injection was 7–10 times higher than that from untreated mice, and the number of NK cells in PyVTu-injected mice was ∼46 times higher than in untreated mice (Fig. 5A). The percentage of γδ T cells did not change after PyVTu cell injection, but the number of γδ T cells in the peritoneum of PyVTu–injected mice was ∼8 times higher than in the untreated controls (Fig. 5A).

NK cells from the PEC of PyVTu cell-injected mice had increased IFNγ production compared to untreated mice, a higher percentage of cells was IFNγ+, and the IFNγ staining had a higher MFI. This difference was also consistent, but smaller in magnitude in the absence of ex vivo PMA and ionomycin stimulation. However, γδ T cells in the PEC did not have similar increases in IFNγ production after PyVTu cell injection (Fig. 5B, C and D). In the same experiments both NK cells and γδ T cells from PEC had increased granzyme-B production after PyVTu cell injection i.p. compared to untreated mice, and this was manifested by higher percentages of granzyme-B positive NK and γδ T cells and higher MFI of granzyme-B staining for NK cells (Fig. 5E, F and G). The IFNγ production was greatly stimulated by a brief in vitro PMA and ionomycin treatment, but granzyme-B production did not require any stimulation in vitro. I.p. injection of RMA cells into mice did not result in an increase in peritoneal NK cells and NK or γδ T cell activation, as judged by intracellular IFNγ and granzyme-B staining (see Fig. S1A and B). This result indicates that the ability to activate NK cells and γδ T cells in the peritoneal cavity is somewhat selective for PyVTu cells, and it does not occur in response to injection of any murine tumor cell line.

**Figure 6:**
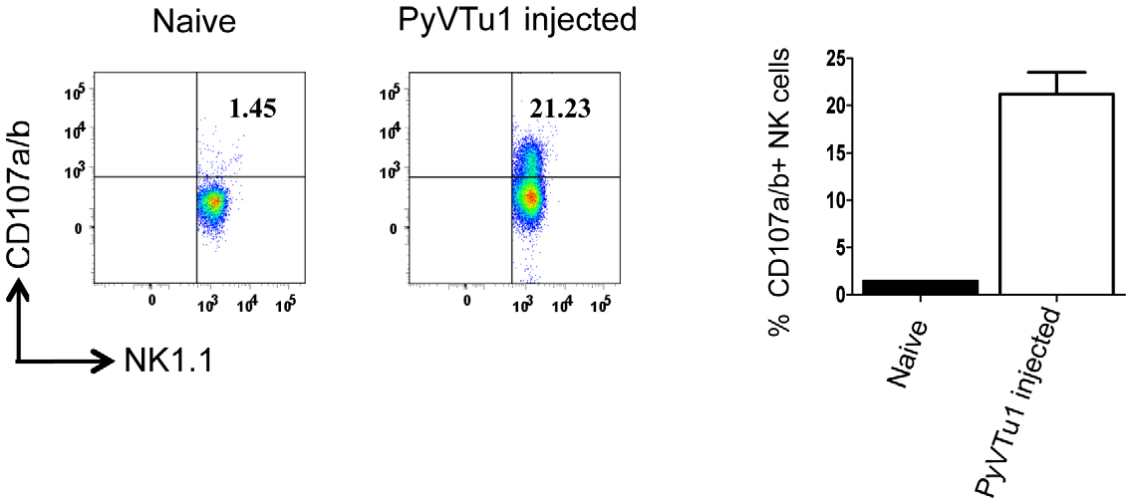
CD107 a/b staining of NK cells from PEC of TCRβ KO mice injected with PyVTu1 cells in-vivo indicates cytotoxic potential. PECs from 4 untreated (pooled) and from 3 i.p. PyVTu1 cell- injected TCRβ KO mice were harvested three days after tumor cell injection and tested for CD107a/b expression by flow cytometry. The cells were gated on NK1.1+ CD3− NK cells.

CD107a and CD107b (LAMP1 and LAMP2) is expressed on the surface of NK cells and CD8 T cells as they undergo activation-induced degranulation, and these markers can be used as a direct measure of the cytotoxic potential of these cells [26]. There was a significant increase in CD107a and CD107b expression on NK cells from PEC of mice injected i.p. with PyVTu cells in comparison to the NK cells from untreated mice (Fig. 6). This result is consistent with our other findings and gives additional support for the notion that PyVTu cells induce cytolytically active NK cells in vivo. Similar experiments performed with γδ T cells did not indicate CD107a or CD107b up-regulation.

PyVTu cell lines harbor a high number of PyV DNA genomes (5×10^8^ copies/µg of cell DNA) and also shed some infectious virus particles. This raises the question whether the activation of NK cells and γδ T cells by PyVTu cells in vivo could be merely due to the released infectious virions. Several lines of experimental evidence suggest that this was not the case. First, i.p. infection of mice with 2×10^6^ p.f.u. of PyV (a virus dose several orders of magnitude higher than the amount of virus which would be shed by injected PyVTu cells) did not result in increases of PEC, NK or γδ T cell percentages or numbers comparable to the ones observed after injection of PyVTu cells (Fig. S2A). Second, PyV infection i.p. activated NK and γδ T cells to produce IFNγ as did PyVTu injection (Fig. S2B), but in contrast to PyVTu cells, the viral infection did not lead to high levels of granzyme-B production (Fig. S2C). Based on these findings we reason that the activation and expansion of NK and γδ T cells by PyVTu cells could not be merely due to infectious virus release by PyVTu cells.

### NK cells kill PyVTu cells by a NKG2D-dependent mechanism

**Figure 7:**
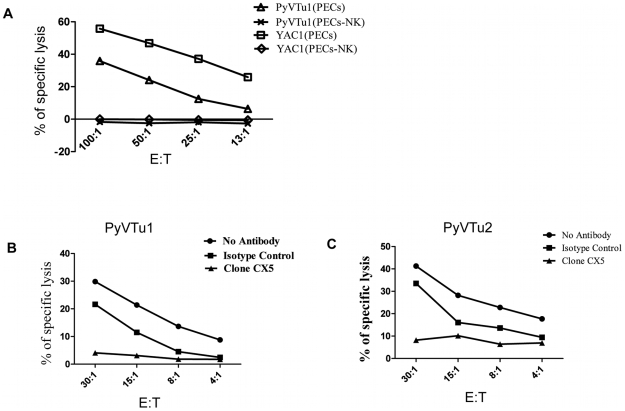
NK cell kill PyVTu targets in a NKG2D-dependent manner. (A) PECs activated in vivo by i.p. injection of PyVTu cells two days prior to harvest were used as effectors, and PyVTu1 and YAC-1 cells were used as target cells in an in vitro Cr release assay. The PECs were derived from TCRβ KO mice or from TCRβ KO mice treated with anti-NK1.1antibodies (PECs-NK). (B and C) NK cells enriched from spleens of uninfected SCID mice were used as effectors against PyVTu1 (B) and PyVTu2 (C) cell targets in the presence of NKG2D blocking (clone CX5) or isotype control antibodies.

**Figure 8:**
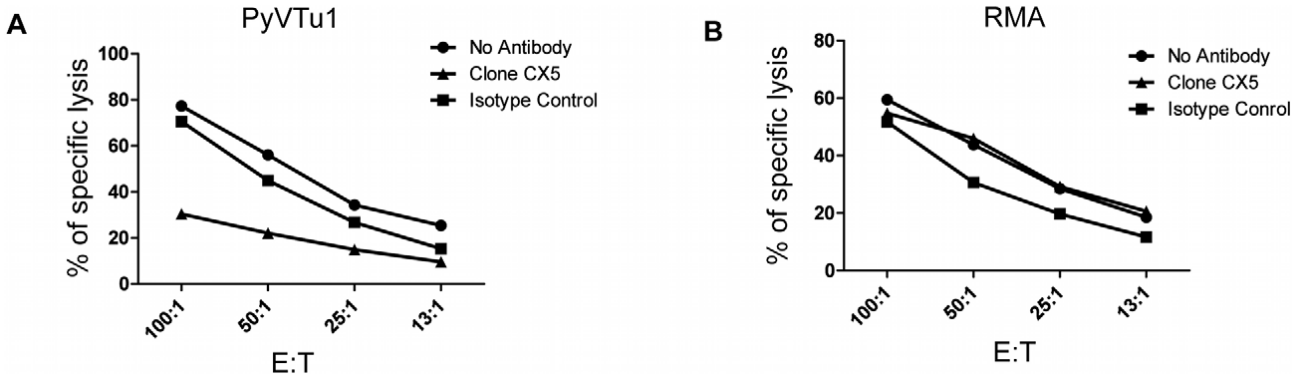
NKG2D blocking antibodies prevent killing of PyVTu cells, but not RMA cells targets by activated NK cells in vitro. In vitro cytotoxicity assays with activated PEC effector cells from TCRβ KO mice and with PyVTu1 (A) and RMA (B) targets in the presence of no antibodies, NKG2D blocking antibody or isotype control antibody. The PEC effectors were activated in vivo by i.p. injection of PyVTu cells two days prior their harvest.

NK cells are thought to exert a potent antitumor activity by directly killing tumor cells. We tested the ability of in vivo activated NK cells to kill PyVTu cells in vitro in chromium release cytotoxicity assays. PyVTu and YAC-1 cell targets were efficiently killed by PEC effectors taken from TCRβ KO mice injected with PyVTu cells three days prior to the PEC harvest. This killing was completely abolished, however, when the PEC was taken from PyVTu-injected, NK cell-depleted mice (Fig. 7A). These results demonstrated that PyVTu cells are sensitive to activated NK cell-mediated killing. NK cells enriched from spleens of untreated SCID mice by MACS separation likewise killed PyVTu targets, and importantly, this killing was completely prevented by treatment with an NKG2D blocking monoclonal antibody CX5, but not by treatment with an isotype control antibody (Fig. 7B and C). Experiments with another NKG2D-specific blocking monoclonal antibody, MI6, gave similar results (data not shown). NK cell-mediated killing of RMA cell targets, on the other hand, was not prevented by CX5 treatment. RMA cells do not express known NKG2D ligands, and therefore they may be recognized and killed by activated NK cells by NKG2D-NKG2D ligand-independent mechanisms (Fig. 8A and B). From these studies we conclude that the interaction of the activating receptor NKG2D on NK cells with NKG2D ligands, such as Rae-1 expressed on PyVTu cells was essential for the NK cell-mediated killing of these virus-induced tumor cells.

## Discussion

In this report for the first time we provide evidence for the critical role of γδ T cells and NK cells in mice in the resistance to naturally occurring virus-induced tumors. Following infection with PyV, a natural mouse pathogen with strong oncogenic potential, mice that lacked αβ T cells, but had γδ T cells, remained tumor free, but mice lacking both αβ and γδ T cells developed tumors by six months post infection. An additional role for NK cells was suggested by an earlier onset of tumor formation in mice lacking both NK and T cells in comparison to T cell-deficient mice that have NK cells.

An increasing body of data obtained in both mice and humans suggests that γδ T cells may constitute an important component of the immunological resistance to tumors, although direct evidence showing that they control tumors induced by viral infections in vivo has been lacking so far. γδ T cells were found among tumor infiltrating lymphocytes (TIL) in human tumors such as lung cancer, colon carcinomas and renal cell carcinomas [1], [2], [3], [27], [28]. Moreover, γδ T cell clones established from TIL or PBMC of cancer patients could kill autologous tumors in vitro in cytotoxicity assays [27], [29], [30], [31], and the growth of human melanoma cells engrafted on SCID mice was inhibited by transfusion of Vδ1 γδ T cells and NK cells from the same patient [32]. Mice deficient in γδ T cells have been shown to have increased susceptibility to chemically induced cutaneous tumors [9], but their role in resistance to natural virus-induced tumor formation had never been demonstrated.

NK cells preferentially kill cells with low MHC class I expression, and tumor cells often down-regulate class I expression to escape from CD8+ T cell responses [33]. Therefore, tumor cell killing was postulated decades ago to be a major function for NK cells [7]. Indeed, low NK cell-mediated cytotoxicity of PBMC correlates with increased risk of tumor development in people [34]. Moreover, NK cell infiltration of tumors is associated with a better prognosis in a variety of carcinomas [4], [5], [6], and transfer of alloreactive NK cells increases the survival of patients with leukemia [35]. Implanted syngeneic tumors grow more aggressively in mice if no functional NK cells are present. Administration of cytokines known to enhance NK cell function can lead to accelerated elimination of implanted tumors [7]. The role of NK and γδ T cells in the control of naturally occurring or spontaneous tumors, however, has been less understood. Here we show data indicating a major role for both γδ T cells and NK cells in tumor immunity in a natural model of tumors induced by virus infection.

NKG2D is a molecule known to be involved in target recognition of both γδ T cells and NK cells [36], it is a type II trans-membrane glycoprotein with a C-type lectin binding domain, expressed as a disulfide-linked homodimer. Its ligands, Rae-1 (α–ε), H60 and Mult-1 in mice, are regarded as markers of cell stress [37], [38]. Here we demonstrate that cell lines established from PyV-induced tumors express Rae-1. These cells activate NK and γδ T cells in vitro and in vivo, and NK cells kill these tumor cells in vitro in an NKG2D-dependent fashion. These findings suggest that γδ T cells and NK cells may recognize emerging PyV-transformed cells expressing Rae-1 stress molecules by their NKG2D receptors. NKG2D is a major activating receptor on NK cells, although NK cell activation is influenced by a variety of other receptors as well. The signals received from NKG2D are transmitted via DAP10, and in mice also the DAP12 signaling molecules [36], [39], [40], [41]. γδ TCR and NKG2D are both major sources of activating signals on γδ T cells. Whether the γδ TCRs get any stimulation from from PyV-induced tumors is an open question.

Freshly isolated γδ T cells lack in vitro cytolytic activity in most studies, and there are only a few reports on in vitro cytotoxic activity using target cells infected by pathogens [42]. In contrast to the easily demonstratable cytotoxic activity mediated by NK cells, we could not demonstrate in vitro killing of PyVTu cells by γδ T cells in this study. Nevertheless, the tumor resistance of TCRβ KO and susceptibility of TCRβ×δ KO mice strongly argues for the involvement of some γδ T cell effector mechanisms in the antitumor responses in vivo.

A variety of virus-infected cells also express Rae-1 and other stress molecules recognized by NKG2D. Examples for the viruses which induce these stress molecules are HCMV, MCMV, influenza A and Epstein Barr Virus [43]. Acutely PyV-infected TCRβ KO mice on day 7 post infection, however, did not show an increase in Rae-1 transcripts in spleen and salivary gland tissues (Fig. S3A). Moreover, in SCID mice on day 6 post infection, when spleens have a high PyV load, macrophages and DC, cell types known to be infected with PyV, did not up-regulate Rae-1 expression (data not shown). Therefore PyV infection of these cells in vivo does not seem to induce Rae-1 expression on their surface. In vitro infection of NIH 3T3, UC1b and MC57G cells with PyV does not change their expression of Rae-1 protein, and only in primary mouse embryonic fibroblast (MEF) cultures was an increase in Rae-1 expression seen after PyV infection (Fig. S3B).

Killing of PyVTu cells by activated NK cells in vitro was prevented by NKG2D blocking antibodies, showing that NKG2D-dependent mechanisms play an essential role in NK cell-mediated cytotoxic responses to PyVTu cells. These findings suggest that γδ T cells and NK cells may control the outgrowth of PyV-induced tumors via NKG2D but would not eliminate permissively infected cells by the same mechanisms. This scenario is supported by our findings that TCRβ KO and TCRβ×δ KO mice did not differ in viral load at various time points post PyV infection. Thus, γδ T cells do not have a significant effect on the control of PyV levels in vivo. Of note is that the titers of T cell-independent antiviral IgG responses are not significantly different in PyV-infected TCRβ KO and TCRβ×δ KO mice, therefore γδ T cells do not contribute as helper cells enhancing antiviral humoral immunity either [44]. Previously we have also reported that NK depletion did not lead to increased viral titers in various organs of PyV-infected SCID mice [24], suggesting that NK cells have no direct antiviral role in these animals. Thus, we conclude that γδ and NK cells seem to mount an antitumor, but not an antiviral response in PyV-infected mice.

PyV can infect a variety of cell types and replicate in a broad range of tissues in mice, and it also has a strong ability to transform cells and induce tumors. Nevertheless, PyV-induced tumors are rare in nature or in wild type adult mice. This suggests that the immune system applies a very successful, multi-pronged strategy against PyV-induced tumor formation. Persisting high virus levels seem to be a prerequisite for tumor formation. Therefore the control of viral spread and replication by B cells and αβ T lymphocytes decreases the likelihood of cell transformation and the generation of tumors. Humoral immunity reduces the viral levels even in the absence of T cell help [15], but B cell responses by themselves do not prevent tumor formation, as TCRβ×δ KO mice containing B cells develop tumors. CD8+ αβ T cells act against virus-infected cells and also against tumor cells expressing viral epitopes [11]. A reduced population of middle T epitope-specific Vβ6+ CD8 αβ T cells in H2k mice carrying Mtv-7 superantigens seems to correlate with increased tumor-susceptibility of these mice when infected as neonates [19]. Our study now shows for the first time, that when αβ T cells are not functional, NK cells and γδ T cells can provide an additional potent line of defense against virus-induced tumor development, by responses that are not specific for viral-coded proteins, but instead probably directed against tumor cell-expressed stress molecules.

The high tumor incidence observed in PyV-infected TCRβ×δ KO mice that have NK cells brings up the question of why NK cells cannot overcome tumor development in the absence of γδ T cells, and why NK cells only delay but don't prevent tumor formation. We speculate that this finding can be explained by the concept of immune editing, which describes the interaction of emerging tumors with the host immune system in three phases, elimination, equilibrium and escape [45]. NK cell responses may represent the first phase of immune editing, eliminating only a fraction of the emerging tumors. The remaining tumor cells eventually may overwhelm the NK cells, perhaps by escaping recognition and/ or loss of NK functionality. This hypothetical scenario is supported by our preliminary findings that large tumors freshly isolated from TCRβ×δ KO mice although express Rae-1 mRNA, lack Rae-1 protein expression on the cell surface (R. Mishra unpublished). Mice which have γδ T cells in addition to NK cells, however, have a numerical advantage of effector cells against the emerging tumors. As a consequence, either all tumor cells may be eradicated at the elimination phase, or if residual tumor cells are left, they may be handled by a second wave of effectors, which may sense and attack the tumor cells, perhaps by another mechanism.

The findings of this study have implications to human cancer. It has been known for decades that most people harbor polyomaviruses, such as BK and JC virus, which persist at low levels, but are harmless in healthy individuals, similar to PyV in normal mice. Patients with impaired immunity, however, suffer from severe pathology associated with the reactivation of these viruses. New human polyomaviruses have recently been identified [46], and one of them is associated with the malignancy Merkel cell carcinoma. This neuroepithelial cancer is rare, and develops mostly in immune-compromised individuals. A large majority of the population is seropositive for the Merkel cell carcinoma-associated polyomavirus and may have a low level persistent infection. New insights obtained in the mouse PyV model may, in addition to elucidating general mechanisms of tumor control, also help to understand and treat polyomavirus –associated human malignancies.

## Materials and Methods

### Mice and infections

All the mice used in the studies were on the C57BL/6 (B6) background. TCRβ KO, TCRβ×δ KO and SCID mice were obtained from the Jackson Laboratory (Bar Harbor, Maine), and colonies of these mice were maintained in the Department of Animal Medicine of the University of Massachusetts under specific pathogen free conditions. E26 mice which express the human CD3E transgene in high copy numbers and are defective in T cells and NK cells [21] were originally obtained on the CBA/J×C57BL/6 mixed background from the Jackson Laboratory, and were fully back crossed onto the C57BL/6 background and bred at the University of Massachusetts Medical School. Mice were used between 8 and 12 wk of age, virus infections were done i.n. or i.p. with 2×10^6^ PFU of PyV strain A2. All the procedures using animals were done according to the protocols “Immunology of virus infections” approved by the University of Massachusetts Medical School Animal Care and Use Committee.

### Quantitative PCR (qPCR) to measure viral DNA genome copy number

DNA was prepared from organ homogenates by digestion with proteinase K (Sigma) at 55°C overnight, followed by phenol extraction and RNase-A treatment (10u/µl, Promega). The PCR amplification was performed as described previously [47]. 50 µl reaction mix containing 50 mM Tris pH 8.0, 0.5 µg/ml BSA, 3 mM MgCl_2_, 0.25 mM of each deoxynucleotide triphosphate, 0.5 U of Taq polymerase (Promega), 0.66 U SYBR-Green (Molecular Probes), 0.1mM each of forward and reverse primer (Invitrogen), 5nM Fluorescein (Bio-Rad) and 1 µg of the DNA sample tested was used. The following primers were used: β actin forward CGA GGC CCA GAG CAA GAG AG; β actin reverse CGG TTG GCC TTA GGG TTC AG; PyV VP1 forward CCC CCG GTA CAG GTT CAG TCC CAT CAT; VP1 reverse GGC ACA ACA GCT CCA CCC GTC CTG CAG. The amplification for VP1 started with one cycle at 95°C for 3 min, 37 cycles of 95°C for 30 sec, 65°C for 20 sec, 72°C for 45 sec. PCR amplification with the β actin primers started with one cycle at 95°C for 150 sec, then 40 cycles of 95°C for 30 sec, 62°C for 25 sec, and 72°C for 25 sec. Negative controls included a sample with no DNA substrate, and DNA from uninfected mouse organs. Three-fold serial dilutions of DNA prepared from uninfected mouse organs (spanning 1µg- 31 ng) were used to generate a standard curve for β actin PCR. For PyV we used a recombinant plasmid containing the VP1 coding sequences and made dilution series from 2×10^8^ copies to 20 copies and mixed these plasmids with 1 µg of DNA from uninfected mouse organs. All the reactions were run in duplicates. The obtained PyV copy numbers were normalized for β actin which reflected the amount of mouse genomic DNA present, and the results were expressed as PyV genome copies/ µg organ DNA.

### Generation of PyV tumor cell lines

Salivary gland tumors were aseptically excised from euthanized PyV-infected TCRβ×δ KO mice. The tumor tissues were rinsed with sterile DMEM containing antibiotics and cut into small pieces and digested with type I collagenase (100U/ml) in 10ml of DMEM containing 10% FCS for 1 hr. The cells were then harvested and plated in DMEM containing 10% FCS. Subsequently the adherent cells were trypsinized and propagated through multiple passages to obtain the transformed cell lines. The three salivary gland tumor cell lines used in these studies, PyVTu1, PyVTu2 and PyVTu3, were independently derived from three tumor bearing TCRβ×δ KO mice. These cell lines have been propagated for over 30 to 40 passages so far without showing major changes in growth or survival.

### Reverse transcriptase (RT) PCR and qRT PCR to detect NKG2D ligand messages

RNA samples from various organs or cell lines were isolated by Trizol (Invitrogen) or using the RNeasy mini kit (Qiagen) following the manufacturer's protocol. Two µg of total RNA from each samples was used to synthesize 1st strand cDNA using 0.5µg of oligo dT (Invitrogen) and superscript II RT (Invitrogen) following the manufacturer's protocol. The PCR amplification was carried out in a total volume of 50µl containing 0.2mM of each dNTP, 0.5 U of Taq polymerase (Invitrogen) in 1× PCR buffer supplied by the manufacturer and 20 pM each of forward and reverse primer (Invitrogen). The following primers were used: β-actin forward primer 5′ CGA GGC CCA GAG CAA GAG AG and β-actin reverse primer 5′ CGG TTG GCC TTA GGG TTC AG; Rae-1 forward primer 5′TGA GCT GGA GAT CAG CTA ATG A and Rae-1 reverse primer 5′ GAA GCG GGG AAG TTG ATG TA; H60 forward primer 5′ CAT GGA GCA GTG GAA GAA CA and H60 reverse primer 5′ CAC TCA GAC CCT GGT TGT CA; Mult1 forward primer 5′ CAA TGT CTC TGT CCT CGG AA and Mult1 reverse primer 5′CTG AAC ACG TCT CAG GCA CT. For qPCR SYBR green master mix (Applied Biosystem) was used. PCR amplification with the β-actin primers started with one cycle at 95°C for 10 minutes, then 37 cycles of 95°C for 30 sec, 62°C for 25 sec, and 72°C for 25 sec. Negative controls included a sample with no DNA substrate. For Rae-1, H60 and Mult1 primers PCR cycles started with 95°C for 10 minutes, then 32 cycles of 95°C for 30 sec, 55°C for 30 sec, and 72°C for 30 sec. For determining relative Rae-1 expression, Rae-1 copy numbers were normalized for β-actin obtained from standard curves.

### Detection of NKG2D L expression by flow cytometry

To stain for NKG2D ligands 2.5×10^5^ to 5×10^5^ PyVTu cells, YAC-1 and RMA cells were treated with anti-CD16/32 (Fc block; clone 2.4G2; BD Pharmingen) and then stained with the following antibodies: PE-anti-mouse Rae-1 (pan-specific, clone 186107), PE-anti-mouse H60 (clone 205326) and Rat IgG_2A_ isotype control-PE (clone 54447) from R&D, PE-anti-mouse MULT-1 (clone 5D10) and Armenian hamster IgG isotype control(clone ebio299Arm) from eBioscience, and PE-anti-mouse MHC class I H-2K^b^ (clone AF6-88.5; BD Bioscience) or class I H-2K^k^ (clone 36-7-5; BD Bioscience).

### NK cell and γδ T cell activation assays, intracellular IFNγ, granzyme-B and CD107a/b staining

For in-vitro assays single cell suspensions were prepared from spleens. Two times 10^6^ spleen leukocytes were incubated with 10^5^ PyVTu cells for 6 hours in 0.2 ml RPMI containing 10% FCS. For stimulation 50 ng/ml of PMA (Sigma) and 500 ng/ml of ionomycin (Sigma) were added after 2 hr of incubation, and parallel cultures were left unstimulated. For the final 3 hours of the incubation time 0.2µl of golgi plug (BD Bioscience) and 0.13 µl of golgi stop (BD Bioscience) was added to allow accumulation of intracellular proteins.

For in vivo assays 5×10^6^ PyVTu cells in HBSS were injected i.p. into TCR β KO mice, control mice were injected i.p. with HBSS with no cells. Three days after injection PEC were harvested from the mice, RBC were removed by lysis and 2×10^6^ of the PEC were incubated for 4 hours in RPMI/ 10% FCS with or without PMA and ionomycin stimulation. Similarly to the in vitro assays, for the final 3 hours of the incubation time 0.2µl of golgi plug (BD Bioscience) and 0.13 µl of golgi stop (BD Bioscience) was added to allow accumulation of intracellular proteins. The cells tested for in vitro or in vivo activation were then treated with anti-CD16/32 (Fc block; clone 2.4G2; BD Pharmingen) and surface stained with FITC-anti-mouse-CD3 (clone- 145-2C11; BD Pharmingen), PerCPCy5.5-anti-mouse NK.1.1 (clone- PK136; BD Pharmingen), and PE-anti-mouse-γδ TCR (clone-GL3; BD Pharmingen) antibodies for 25 minutes at room temperature. Cells were then washed and permeabilized with Cytofix/Cytoperm buffer (BD Biosciences), followed by staining with PE Cy7-anti-mouse-IFNγ (clone XMG1.2; BD Pharmingen) and APC-anti-human-granzyme-B (clone GB11; Invitrogen) for 20–25 minutes at room temperature. For CD107 staining both FITC-anti-mouse-CD107a (clone-1D4B; BD Pharmingen) and FITC-anti-mouse-CD107b (clone-ABL-93; BD Pharmingen) antibodies were added along with golgi plug and golgi stop for four hours and then treated with anti-CD16/32 (Fc block; clone 2.4G2; BD Pharmingen) and surface stained with PerCPCy5.5-anti-mouse NK.1.1 (clone PK-136; BD Pharmingen), PE-anti-mouse-γδ TCR (clone GL3; ) for 25 minutes. The cells were finally analyzed by flow cytometry.

### In vitro cytotoxicity assays

Standard 4 h ^51^Cr release microcytotoxicity assays were used to determine NK cell activity [48]. Activated PECs or spleen cells of SCID mice were used as effector cells, in some experiments the NK cells were enriched by using an NK cell isolation kit (MACS, Miltenyi Biotech) following the manufacturer's protocol. The PECs were activated in vivo by an injection of 4×10^6^ to 5×10^6^ PyVTu cells i.p. two days prior to their harvest. ^51^Cr-labelled YAC-1 cells, PyVTu cells or RMA cells were used as targets, and 10^4^ target cells were plated into wells of microtiter plates with varying numbers of effectors to achieve the planned effector to target (E∶T) ratios. After 4 hours of incubation ^51^Cr release into the supernatants was measured. The percentage of specific ^51^Cr release was calculated as described before [42]. For NKG2D blocking, anti-NKG2D blocking antibodies (clone CX5; eBioscience or clone MI-6; eBioscience ) or corresponding isotype controls (Rat IgG1 isotype HRPN; BioXcell and Rat IgG2a isotype eBioscience) were used at 5µg/ml. For depletion of NK cells anti-NK1.1 antibody (clonePK136; BioXcell ) was injected at the dose of 40 µg/mouse i.p. one day prior to injection of PyVTu cells.

## Supporting Information

Figure S1 NK and γδ T cells are activated after i.p. injection of PyVTu cells but not Rae-1 negative RMA cells. (A) Intracellular IFNγ and granzyme-B staining of NK cells and (B) γδ T cells isolated from the peritoneal cavity of TCRβ KO mice that received i.p. injection of PyVTu1 cells or RMA cells three days prior their harvest. The cells were tested for IFNγ with or without in vitro PMA and ionomycin stimulation.
(1.88 MB TIF)

Figure S2 Activation of NK cells and γδ T cells in vivo by i.p. injection of PyVTu cells or PyV. (A) Left Panel: Increase in peritoneal NK and γδ T cells in response to i.p. injection of PyV (2×10^6^ p.f.u.) or PyVTu1 cells (5×10^6^). PEC harvested from mice (n = 3) 3 days after injection were analyzed individually by flow cytometry. The numbers show percentages of NK1.1+/CD3− NK cells and γδ TCR+/CD3+ γδ T cells, respectively. Right panel: mean + sd of NK and γδ T cell numbers in the PECs of PyV-infected and PyVTu cell-injected mice in the same experiment. (B) Intracellular IFNγ and (C) granzyme-B staining of cells harvested from the peritoneal cavity of mice three days after i.p. injection of PyV or PyVTu cells, gated on NK (upper panels) and γδ T cells (middle panels). IFNγ and granzyme-B production was tested with or without in vitro PMA and ionomycin stimulation. The numbers indicate IFNγ + or granzyme-B + cells, respectively. Bottom panels: percentages and MFI of IFNγ+ and granzyme-B+ NK and γδ T cells. Filled bars represent pooled samples from 2 PyV-infected mice without stimulation, open bars the means and s.d. of 3 PyVTu-injected mice without stimulation, the bars with horizontal stripes pooled samples from 2 PyV-infected mice with stimulation and the bars with vertical stripes the means and s.d. of 3 PyVTu-injected mice with stimulation.
(1.70 MB TIF)

Figure S3 Acute PyV infection does not induce Rae-1 mRNA and protein expression in vivo in TCRβ KO mice, or in tissue culture. (A) Relative Rae-1 expression measured by qPCR in the spleens and salivary glands of naïve and 7 day PyV- infected TCRβ KO mice and in PyVTu cell lines. N = 3 for both naïve and infected tissue samples; for tumor cell lines average of PyVTu1, PyVTu2 and PyVTu3 is shown. (B) Expression of Rae-1 protein in primary mouse embryonic fibroblast cells, and NIH3T3, UC1B and MC57G cell lines uninfected or PyV-infected for three days at a MOI of 1. The open box in each case shows the uninfected isotype control antibody treated cells, the light shaded grey box represents the Rae-1-specific antibody- stained uninfected cells and the dark shaded grey box represents Rae-1 specific antibody- stained PyV- infected cells.
(0.95 MB TIF)
