# Supplementary figures and images for: NK Cells and γδ T Cells Mediate Resistance to Polyomavirus–Induced Tumors
Source: PLoS Pathog. 2010 May 27;6(5):e1000924. doi: 10.1371/journal.ppat.1000924 (PMC2877738; doi:10.1371/journal.ppat.1000924)

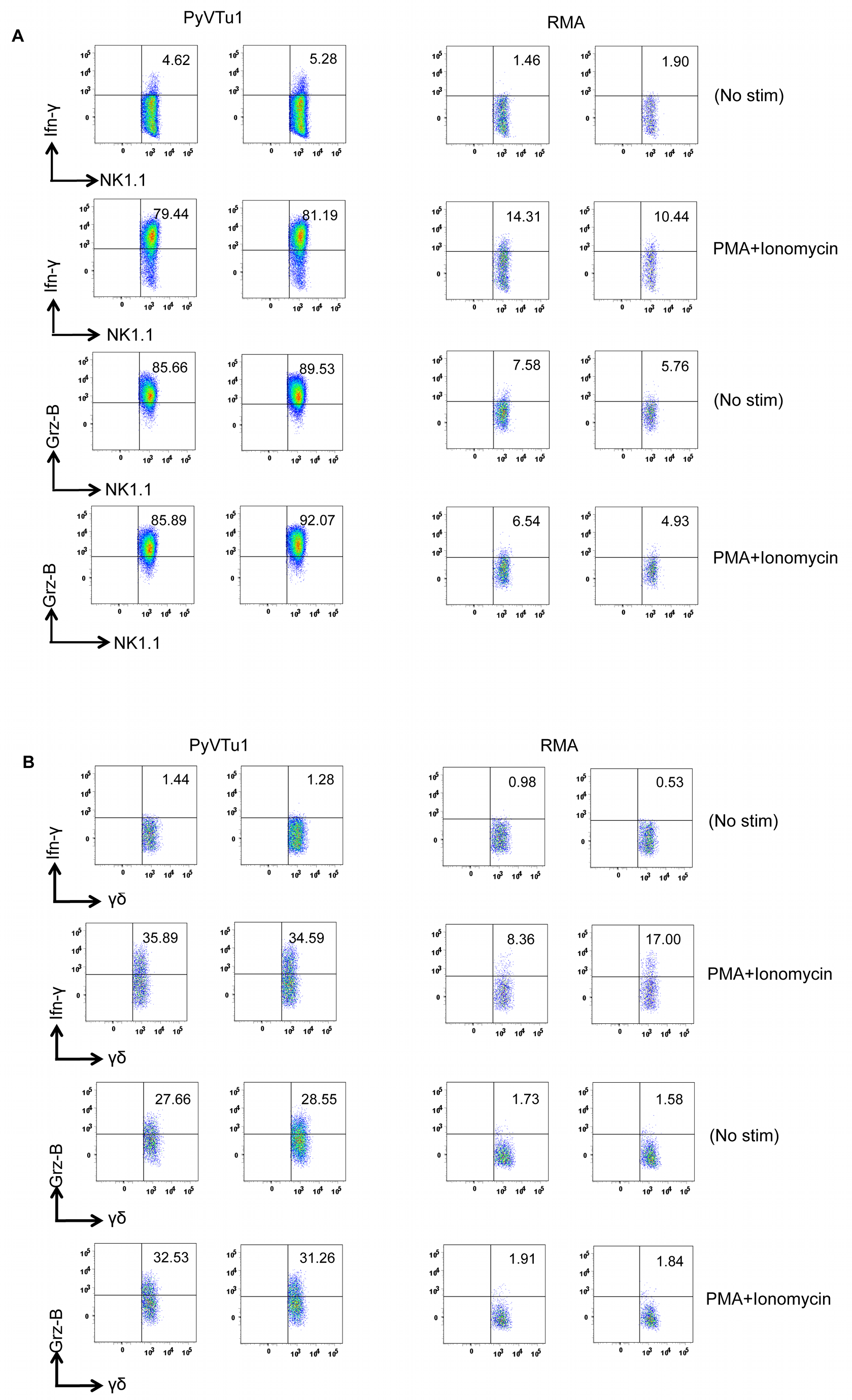

Supplement: Figure S1 — NK and γδ T cells are activated after i.p. injection of PyVTu cells but not Rae-1 negative RMA cells. (A) Intracellular IFNγ and granzyme-B staining of NK cells and (B) γδ T cells isolated from the peritoneal cavity of TCRβ KO mice that received i.p. injection of PyVTu1 cells or RMA cells three days prior their harvest. The cells were tested for IFNγ with or without in vitro PMA and ionomycin stimulation. (1.88 MB TIF) [file ppat.1000924.s001.tif]

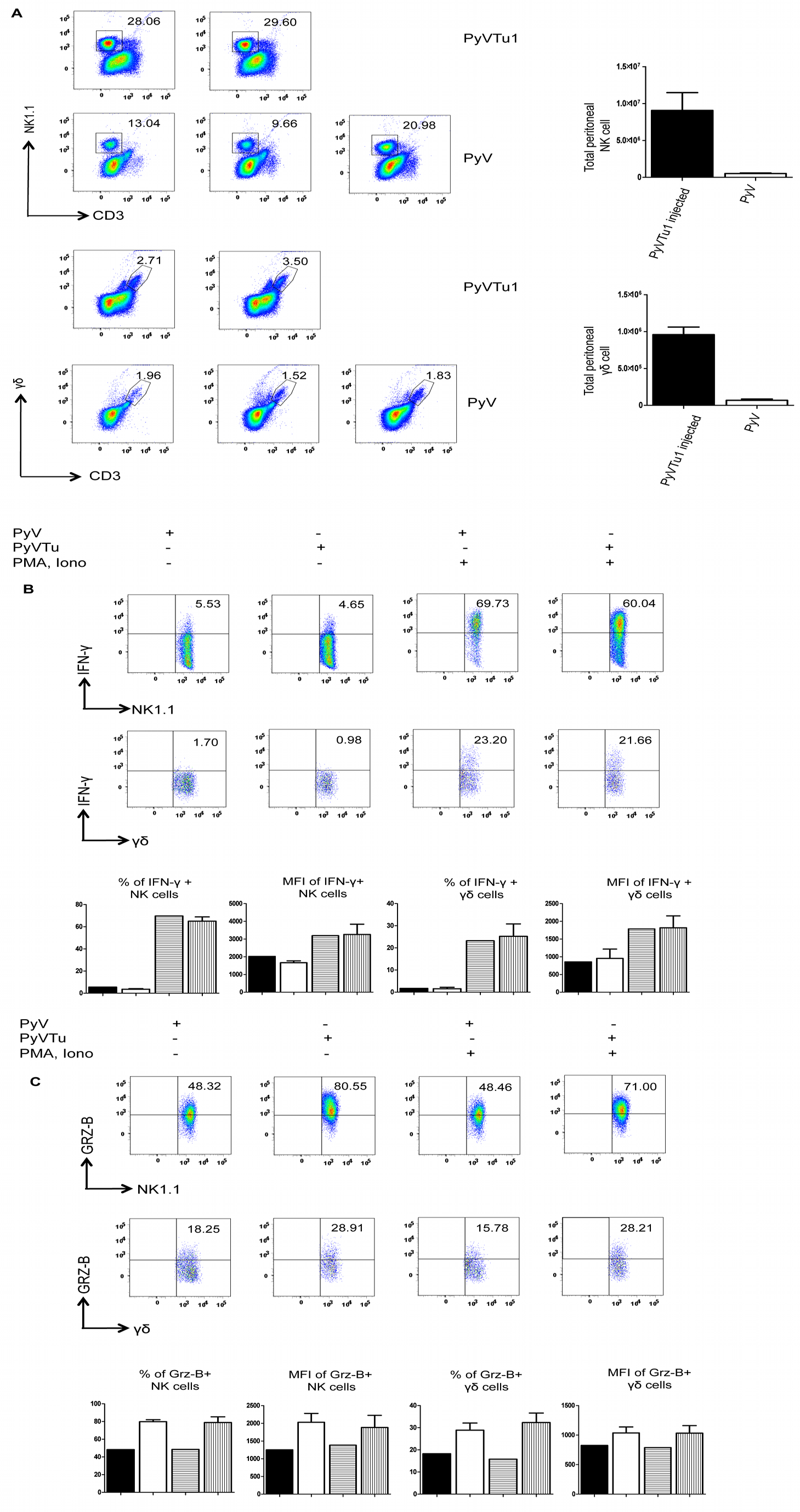

Supplement: Figure S2 — Activation of NK cells and γδ T cells in vivo by i.p. injection of PyVTu cells or PyV. (A) Left Panel: Increase in peritoneal NK and γδ T cells in response to i.p. injection of PyV (2×10^6^ p.f.u.) or PyVTu1 cells (5×10^6^). PEC harvested from mice (n = 3) 3 days after injection were analyzed individually by flow cytometry. The numbers show percentages of NK1.1+/CD3− NK cells and γδ TCR+/CD3+ γδ T cells, respectively. Right panel: mean + sd of NK and γδ T cell numbers in the PECs of PyV-infected and PyVTu cell-injected mice in the same experiment. (B) Intracellular IFNγ and (C) granzyme-B staining of cells harvested from the peritoneal cavity of mice three days after i.p. injection of PyV or PyVTu cells, gated on NK (upper panels) and γδ T cells (middle panels). IFNγ and granzyme-B production was tested with or without in vitro PMA and ionomycin stimulation. The numbers indicate IFNγ + or granzyme-B + cells, respectively. Bottom panels: percentages and MFI of IFNγ+ and granzyme-B+ NK and γδ T cells. Filled bars represent pooled samples from 2 PyV-infected mice without stimulation, open bars the means and s.d. of 3 PyVTu-injected mice without stimulation, the bars with horizontal stripes pooled samples from 2 PyV-infected mice with stimulation and the bars with vertical stripes the means and s.d. of 3 PyVTu-injected mice with stimulation. (1.70 MB TIF) [file ppat.1000924.s002.tif]

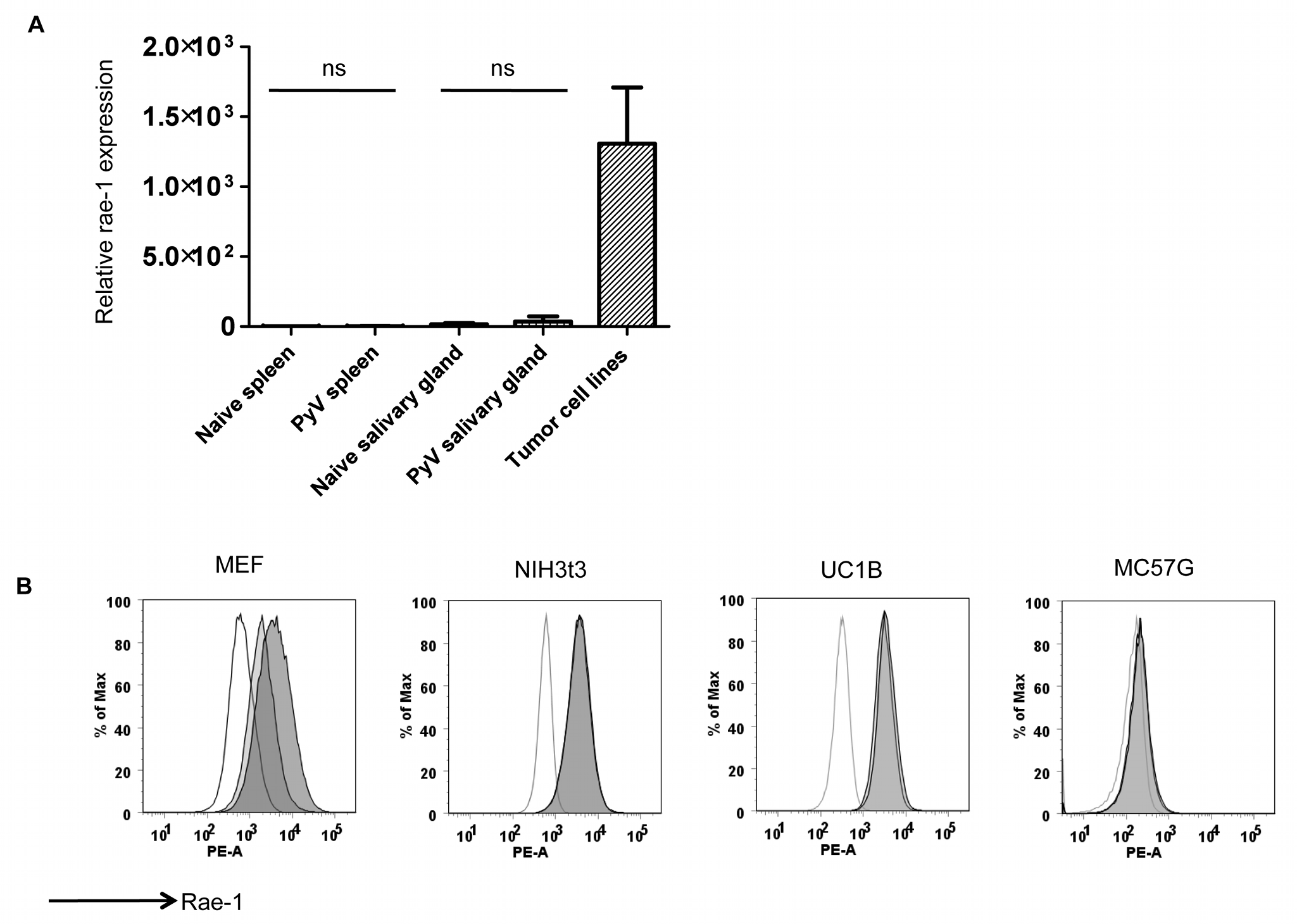

Supplement: Figure S3 — Acute PyV infection does not induce Rae-1 mRNA and protein expression in vivo in TCRβ KO mice, or in tissue culture. (A) Relative Rae-1 expression measured by qPCR in the spleens and salivary glands of naïve and 7 day PyV- infected TCRβ KO mice and in PyVTu cell lines. N = 3 for both naïve and infected tissue samples; for tumor cell lines average of PyVTu1, PyVTu2 and PyVTu3 is shown. (B) Expression of Rae-1 protein in primary mouse embryonic fibroblast cells, and NIH3T3, UC1B and MC57G cell lines uninfected or PyV-infected for three days at a MOI of 1. The open box in each case shows the uninfected isotype control antibody treated cells, the light shaded grey box represents the Rae-1-specific antibody- stained uninfected cells and the dark shaded grey box represents Rae-1 specific antibody- stained PyV- infected cells. (0.95 MB TIF) [file ppat.1000924.s003.tif]
